# New Insights into the Multifaceted Role of Myeloid-Derived Suppressor Cells (MDSCs) in High-Grade Gliomas: From Metabolic Reprograming, Immunosuppression, and Therapeutic Resistance to Current Strategies for Targeting MDSCs

**DOI:** 10.3390/cells10040893

**Published:** 2021-04-14

**Authors:** Senthilnath Lakshmanachetty, Joselyn Cruz-Cruz, Eric Hoffmeyer, Allison P. Cole, Siddhartha S. Mitra

**Affiliations:** Morgan Adams Brain Tumor Research Program, Department of Pediatrics, Division of Hematology/Oncology/Bone Marrow Transplant, University of Colorado Anschutz Medical Campus, Aurora, CO 80045, USA; senthilnath.lakshmanachetty@cuanschutz.edu (S.L.); Joselyn.cruzcruz@cuanschutz.edu (J.C.-C.); eric.hoffmeyer@cuanschutz.edu (E.H.); ALLISON.P.COLE@CUANSCHUTZ.EDU (A.P.C.)

**Keywords:** MDSCs, glioma, glioblastoma, high-grade glioma, brain tumors, metabolic reprogramming, immune suppression, therapeutic resistance, therapeutic targeting, immunotherapy, tumor microenvironment

## Abstract

Cancer cells “hijack” host immune cells to promote growth, survival, and metastasis. The immune microenvironment of high-grade gliomas (HGG) is a complex and heterogeneous system, consisting of diverse cell types such as microglia, bone marrow-derived macrophages (BMDMs), myeloid-derived suppressor cells (MDSCs), dendritic cells, natural killer (NK) cells, and T-cells. Of these, MDSCs are one of the major tumor-infiltrating immune cells and are correlated not only with overall worse prognosis but also poor clinical outcomes. Upon entry from the bone marrow into the peripheral blood, spleen, as well as in tumor microenvironment (TME) in HGG patients, MDSCs deploy an array of mechanisms to perform their immune and non-immune suppressive functions. Here, we highlight the origin, function, and characterization of MDSCs and how they are recruited and metabolically reprogrammed in HGG. Furthermore, we discuss the mechanisms by which MDSCs contribute to immunosuppression and resistance to current therapies. Finally, we conclude by summarizing the emerging approaches for targeting MDSCs alone as a monotherapy or in combination with other standard-of-care therapies to improve the current treatment of high-grade glioma patients.

## 1. Introduction

Malignant gliomas that originate from glial, neural stem cells and astrocytes are the most aggressive tumors of the central nervous system (CNS) and spinal cord with a median survival of less than 12–15 months [1]. The current standard of care therapies such as surgery, radiotherapy, and chemotherapy have had only limited success in increasing the lifespan of glioma patients [2]. Although recent advances in immune checkpoint blockade (ICD) therapies such as anti-PD-1/PD-L1 and anti-CTLA4 have yielded promising results in melanoma and non-small lung cancer [3], glioma patients not only failed to respond in clinical trials but also developed resistance to ICB in a multitude of ways [4]. One such way is the development and maintenance of an immune-suppressive tumor microenvironment (TME) that thwarts the efficacy of existing therapies and host anti-tumor immune responses.

Extensive analysis of the immune microenvironment in high-grade glioma (HGG) using single-cell RNA-seq, mass cytometry (CyTOF), immunohistochemistry, flow cytometry, and other “omics” technologies indicate the presence of higher numbers of immune-suppressive macrophages, microglia dendritic cells, regulatory T-cells, and myeloid-derived suppressor cells (MDSCs) [5]. Together, these cells interact with the neoplastic cells to promote tumor growth, progression, metastasis, angiogenesis and contribute to the extreme immunosuppression observed in HGG.

In healthy humans and mice, MDSCs are present at very low frequencies and constitute only ~0.5–2% of peripheral blood mononuclear cells [6]. Nevertheless, 30–50% of the tumor mass in HGGs are found to be MDSCs [7,8]. Originally, derived from the bone marrow, MDSCs are a very heterogeneous population of immature myeloid cells (IMCs) present at various stages of myelopoiesis. Under normal conditions, IMCs can be differentiated into macrophages, granulocytes, and dendritic cells. However, in pathological conditions such as HGG, the differentiation of IMCs is subverted, resulting in the generation, recruitment, expansion, and activation of MDSCs [9] not only in the tumor bed but also in the peripheral blood [10,11].

Recently, there is a great deal of interest to identify, quantify, characterize, and target the different MDSC populations in brain tumors. In this review, we aim to provide an overview regarding the origin, characterization, and metabolic reprogramming of MDSCs. Moreover, we illustrate the mechanisms by which MDSCs contribute to immunosuppression and resistance to existing therapies. Finally, we conclude by discussing the current strategies and clinical trials that are being pursued to effectively target MDSCs in the setting of high-grade glioma.

## 2. History, Origin, and Characterization of MDSCs in Mice and Humans

Under non-pathological conditions, myelopoiesis is a tightly controlled process by which the body can effectively protect itself from the insult. Conversely, under chronic inflammatory conditions or neoplasia, the immune system cannot keep up with the demand for neutralization as a result leading to deregulated myelopoiesis. One subpopulation of cells which expands prodigiously under such conditions is myeloid-derived suppressor cells (MDSCs). 

In the late 1970s, the presence of an immune-suppressive subpopulation of myeloid cells was first reported in mice following myeloablative radiation therapy [12]. Initially, these cells were referred to as natural suppressor (NS) cells since they did not express any markers related to macrophages, T-cells or B-cells, however, they shared similar characteristics as natural killer (NK) cells [13]. Nearly 20 years later, this population of “suppressor cells” was reported ex vivo in the peripheral blood of patients following cytokine mobilization and apheresis [14]. Around this time, the first characteristic surface antigens of “suppressor cells” derived from the spleen of mice were identified to be Mac-1 and Gr-1 [15]. In 2007, it was proposed that this population be referred to as MDSCs to reflect their shared origin and function, and to lessen confusion in this growing field of interest [16].

**Figure 1:**
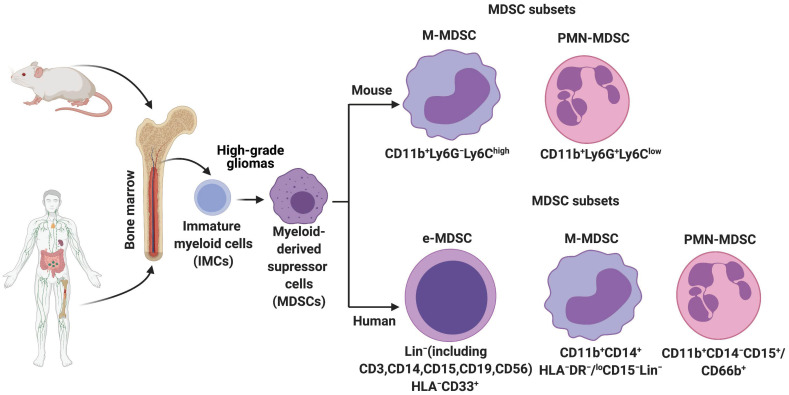
Origin and subsets of myeloid-derived suppressor cells (MDSCs) in mice and humans. Under normal conditions, immature myeloid cells (IMCs) derived from the bone marrow differentiate into Monocytes, Neutrophils, Macrophages, and Dendritic cells. However, pathological conditions such as high-grade gliomas prevent IMC differentiation, resulting in the generation and accumulation of different MDSC subsets. Phenotypic markers that are commonly used to identify these subsets in mice and humans are described.

In HGGs, MDSCs are derived from the immature myeloid progenitors present in the bone marrow (Figure 1). More recently, single-cell RNA-sequencing (scRNA-seq) analysis on mouse breast tumors suggests that abnormal differentiation of monocyte and neutrophil-like cells in the spleen can lead to the generation of MDSCs [17]. Reprogramming or activation of monocytes and granulocytes by exposure to Toll-like receptors, IL-10, WNT5A, LPS, and INFγ can also give rise to MDSCs [18]. Lastly, MDSCs can be generated and activated ex vivo by the addition of GM-CSF, G-CSF, IL-6, and IL-10 to bone marrow precursors obtained from healthy individuals and detailed functional and phenotypic characterization revealed these bone marrow-derived MDSCs (BM-MDSCs) to be similar to the MDSCs present in different cancer patients [19,20].

MDSCs in mice are usually identified by the surface expression of CD11b and Gr1. Further, based on the surface expression, density, and morphology, two subsets of MDSCs are identified: Monocytic (M-MDSC) and polymorphonuclear (PMN-MDSC) or granulocytic MDSC (G-MDSC) [21]. As the name suggests, M-MDSCs are mononuclear cells, whereas PMN-MDSCs are polymorphonuclear cells. Moreover, M-MDSCs are very similar to inflammatory monocytes, and PMN-MDSCs shared some similar characteristics with neutrophils [22]. Irrespective of these similarities, MDSC subsets are a very distinct population and are phenotypically distinguished by the expression of CD11b^+^Ly6G^−^Ly6C^high^ (M-MDSC) or CD11b^+^Ly6G^+^Ly6C^low^ (PMN-MDSCs) (Figure 1) [23].

MDSCs in humans are generally defined as CD33^+^ major histocompatibility class II^−^ or HLA-DR^−^ cells. Further, expression of CD115, CD124, CD80, programmed death ligand-1 (PD-L1), ARG1, and iNOS has been proposed to identify MDSCs [24]. In humans, three subsets of MDSCs exist: Early-stage MDSC (e-MDSC), monocytic MDSC (M-MDSC), and polymorphonuclear (PMN-MDSC) or granulocytic MDSCs (gMDSCs) (Figure 1) [25]. Phenotypic markers used to identify them are lin^−^ (including CD3, CD14, CD15, CD19, and CD56) HLA-DR^−^CD33^+^ (e-MDSCs), CD11b^+^CD14^+^CD15^−^ (M-MDSC), and CD11b^+^CD14^+^CD66b^+^ (PMN-MDSC) (Figure 1) [22]. Furthermore, expression of Lectin-type oxidized LDL receptor 1 (LOX-1), which is associated with ER stress and lipid metabolism, is specifically seen in PMN-MDSC [26]. Aside from the phenotypic markers, suppression of T-cells and reactive oxygen species (ROS) production are also used to functionally characterize MDSCs [20]. M-MDSCs rely on increased nitric oxide (NO) and inducible NO synthase, whereas PMN-MDSCs are dependent on reactive oxygen species (ROS). NO reacts with superoxide in the TME to create peroxynitrite (PNT) which then adds nitrate to T-cell receptors thereby reducing T-cell affinity. On the other hand, ROS production is required for inducing antigen-specific T cell tolerance [27]. Despite these established phenotypic and functional characterization, the accurate identification of different MDSC subsets in mouse and human tumors remains a challenge to date.

## 3. MDSCs in High-Grade Gliomas

Gliomas are classified into four different histopathologic grades: Low-grade gliomas (LGG) (WHO grade I and II) and high-grade gliomas (HGG) (WHO grade III and IV) [28,29]. LGG are rare, occur mostly in children, and commonly have mutations in BRAF, FGR1, ATRX, IDH1, and IDH2 [30]. On the contrary, HGGs are brain tumors that primarily affect adults and have mutations in H3F3A G34R, TP53, EGFR, PTEN, IDH1, and IDH2. The most common and aggressive grade IV, glioblastoma (GBM) typically does not have IDH mutations, nonetheless, frequent intratumoral MGMT promoter methylation is found in these patients [31]. In general, LGG have a lower number of tumor-infiltrating immune suppressive cells compared to HGG [32].

In rat glioma models, an increase in the percentage of MDSCs in the peripheral blood has been reported [33]. Furthermore, it can also serve as a biomarker for tumor recurrence [33]. An increase in MDSCs is also reported in syngeneic and xenograft mouse models of glioma [34]. Corroborating these findings, in humans, elevated levels of MDSCs in the peripheral blood of glioblastoma (GBM) patients have been observed [35], however, this increase is yet to be correlated with increased MDSCs at the glioma site. One possible explanation for this is attributed to the existence of different subsets of MDSCs. In fact, Gielen et al. reported that although both M-MDSCs and PMN-MDSCs are increased in the peripheral blood of GBM patients, PMN-MDSCs and not M-MDSCs are increased in the GBM TME [36]. Conversely, in mouse GBMs, M-MDSCs and not PMN-MDSCs are enriched at the tumor site [37]. Thus, the predominant type of MDSCs present in the TME of HGG is still not clear. Adding to this complexity, Bayik et al. reported that sexual dimorphism can determine which type of MDSC subsets expand and contribute to immune suppression in GBM patients. For instance, higher frequencies of M-MDSCs found in male GBM patients promote tumor progression, whereas increased PMN-MDSCs in female patients prevents anti-tumor immunity and is associated with poor prognosis [38]. In agreement with these findings, in mouse GBM models, depletion of PMN-MDSCs with anti-LY6G antibody only prolongs the survival of female and not male mice. Similarly, reduction of M-MDSC frequencies with fludarabine increases the survival of male and not female mice [38].

**Figure 2:**
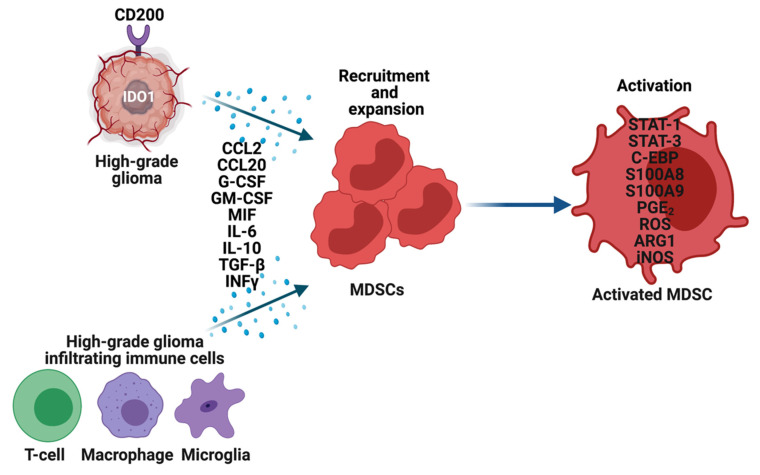
Recruitment, expansion, and activation of MDSCs in high-grade gliomas (HGG). Glioma cells, T-cells, Macrophages, and Microglia in the tumor microenvironment overexpress multiple genes and secrete an array of cytokines, chemokines, and other factors to recruit and expand MDSCs. These factors also activate MDSCs through various mechanisms, which then perform their immune-suppressive functions in HGG.

The precise mechanisms by which MDSCs traffic from bone marrow to the gliomas are not known. To recruit MDSCs from the bone marrow, glioma cells overexpress CD200 [39], indoleamine 2,3-dioxygenase (IDO1) [40] and secrete CCL20 [41], macrophage migration inhibitory factor (MIF) [37], in addition to other growth factors. Along with glioma cells, immune cells such as glioma-associated microglia and macrophages (GAMs) secrete CCL2 to recruit regulatory T-cells (Tregs) and MDSCs (Figure 2). Furthermore, the hypoxic tumor environment upregulates the expression of vascular endothelial growth factor (VEGF) and hypoxia-inducible factor-1 alpha (HIF1α) in glioma cells, which then induces ectonucleoside triphosphate diphosphohydrolase 2 (ENTPD2) and aids in the accumulation of MDSCs by converting extracellular ATP to 5′-AMP [42]. In addition, hypoxia recruits CX3CR1 expressing MDSCs by increasing the expression of CCL26 on tumor cells [43]. Finally, in vitro exposure of MDSCs from the spleen to hypoxia results in their differentiation into immunosuppressive macrophages [44].

Once MDSCs are recruited to the HGG site their expansion and activation are tightly controlled by cytokines (IL-6, IL-10, TGF-β, M-CSF, GM-CSF, INFγ), chemokines (CCR2, and other factors (VEGF) secreted by tumor cells, T cells, microglia, and macrophages (Figure 2) [9,33,45]. Glioma-derived exosomes also induce the expansion of MDSCs by transferring miR-29a and miR-92a [46]. Briefly, the above-mentioned factors and exosomes activate Janus tyrosine kinase (JAK), signal transducer and activator of transcription 3 (STAT3), STAT1, STAT6, CCAAT/enhancer-binding protein (C/EBPs), S100A8, S100A9, prostaglandin E2 (PGE2) in MDSCs to promote their survival, expansion, and function [32,33]. Furthermore, activated C/EBPs promote the expression of immune suppressive arginase 1 (ARG1), reactive oxygen species (ROS), and inducible nitric oxide synthase (iNOS) in MDSCs [47]. Higher levels of arginase activity and G-CSF levels in the peripheral blood of GBM patients have been used as an indicator for the activation and immune suppressive functions of MDSCs (Figure 2) [48].

In summary, glioma and immune cells develop and maintain immunosuppressive TME by recruiting, expanding, and activating MDSCs from the bone marrow and spleen into the peripheral blood, as well as at the tumor site.

## 4. Metabolic Reprogramming of MDSCs

Cancer cells use glycolysis to generate ATP even under aerobic conditions (Warburg effect). This altered metabolism compared to normal cells was first reported by Otto Warburg in 1930 [49]. Over the last few decades, metabolic reprogramming has emerged as a new hallmark that cancer cells use to evade growth suppression and cell death/apoptosis [50]. To this end, they preferentially utilize glycolysis, amino acid metabolism, pentose-phosphate pathway (PPP), and tricarboxylic acid (TCA) cycle to thrive under the harsh environmental conditions presented by the TME [51].

**Figure 3:**
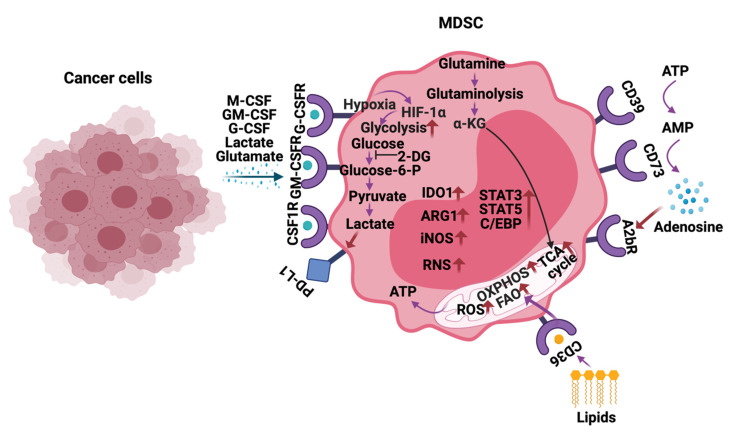
Metabolic reprogramming of MDSCs in the tumor microenvironment. In response to the cytokines and metabolites secreted by the cancer cells, MDSCs alter their metabolism by increasing glycolysis, glutamine/glutaminolysis, fatty acid oxidation (FAO), oxidative phosphorylation (OXPHOS), tricarboxylic acid (TCA) cycle, reactive oxygen species (ROS), and expression of ectonucleotidases CD39/CD73. Additionally, metabolites such as lactate, lipids glutamate, and adenosine in the tumor microenvironment (TME) also play a vital role in regulating the immune-suppressive functions of MDSCs in HGG. The arrows in red indicate activation/upregulation.

Recently, the critical role of metabolism in controlling the functions of immune cells is becoming greatly appreciated [52,53]. Along those lines, increased glycolysis, ROS, fatty acid oxidation (FAO), glutamine metabolism, oxidative phosphorylation (OXPHOS), lipid uptake, and extracellular adenosine are observed in MDSCs infiltrating into the tumors, including HGG (Figure 3) [54]. MDSCs exhibit these metabolic changes to support their development, survival, differentiation, activation, and immunosuppressive activity. Thus, inhibitors targeting the above-mentioned metabolic pathways are being actively explored to block the MDSC activity and improve anti-cancer immunotherapies.

Increased glycolysis in tumor cells makes them secrete cytokines such as M-CSF and GM-CSF into the TME [55]. These cytokines bind to CSF1R and GM-CSFR on MDSCs and upregulate glycolysis, glucose, amino acid uptake, and ATP generation [56,57,58]. Furthermore, glycolysis in tumor cells indirectly supports MDSC development and function by increasing the expression of CCAAT/enhancer-binding protein (CEBP), liver-enriched activator protein (LAP), AMP-activated protein kinase (AMPK)-ULK1, and autophagy pathways [56]. Importantly, inhibiting glycolysis using 2-deoxy-D-glucose (2-DG) decreased the levels of IDO in MDSCs and enhanced their apoptosis [55]. In addition to the tumor-derived factors, hypoxic TME induces HIF-1α dependent glycolysis in MDSCs and promotes their differentiation into immune suppressive tumor-associated macrophages (TAMs) [59]. Moreover, hypoxia also directs the oxidation of pyruvate to lactate in tumor cells. The lactate produced and secreted by tumor cells increases the frequencies of MDSCs and causes the differentiation of tumor-infiltrating neutrophils into MDSCs. Lactate generated in MDSCs via glycolysis increases their programmed cell death-ligand 1 (PD-L1) expression, which then suppresses the T-cell activity through interaction with programmed cell death protein (PD-1) and cytotoxic T lymphocyte-antigen associated protein 4 (CTLA-4) [60]. In line with these findings, inhibition of Lactate dehydrogenase-A (LDH-A) reduces the number of MDSCs arising from spleens in tumor-bearing mice. Similar results were also observed in human cancer patients [61].

Upon infiltrating into the tumor, both M-MDSCs and PMN-MDSCs prefer lipid uptake and fatty acid oxidation (FAO) and partake in tricarboxylic acid (TCA) cycle and oxidative phosphorylation (OXPHOS) in the mitochondria to generate ATP [62]. MDSCs achieve this metabolic switch by increasing CD36/FAT (fatty acid translocase) scavenging receptor, fatty-acid transport proteins (FATP), FAO enzymes (carnitine palmitoyltransferase (CPT1) and 3-hydroxy acyl-COA dehydrogenase (HADHA)), mitochondrial and oxygen consumption, and JAK-STAT (STAT3 and STAT5) signaling [54,63]. Furthermore, continuous ER stress and ROS production as a result of FAO induce the immune suppressive function of MDSCs by increasing the expression of iNOS, ARG1, reactive nitrogen species (RNS), and NADPH oxidase 2 (NOX2). In agreement with these results, pharmacologic inhibition of FATP and FAO blocked immune-suppressive functions of MDSCs and enhanced immune checkpoint therapies [62,64].

Glioma stem cells metabolize glutamine and secrete high levels of glutamate into the TME to evade immune surveillance. A higher concentration of glutamate enables the maturation and infiltration of MDSCs to the glioma site [65]. Glutamine-derived α-ketoglutarate (α-KG) aids in the expansion of MDSCs via the NDMA receptors [66]. The mechanisms by which α-KG regulates MDSCs in HGG are not known. Recent studies suggest that blocking glutamine/glutaminolysis metabolism prevents the recruitment, generation, and metabolic reprogramming of MDSCs by inhibiting tumor-derived G-CSF secretion and promotes the generation of anti-tumor inflammatory macrophages [67].

MDSCs express high levels of the ectonucleotidases CD39 and CD73 and release immunosuppressive adenosine into the TME. TGF-β-mTOR-HIF1-α signaling axis induces the expression of CD39/CD73 on MDSCs in the peripheral blood and tumor tissues [68]. CD39/CD73 expression on MDSCs performs their suppressive activity by inhibiting NK and T-cells via paracrine signaling. Consistent with these observations, pharmacological activation of AMP-activated protein kinase α using metformin caused the inhibition of HIF-1α induced CD39/CD73 expression on MDSCs [69]. Extracellular adenosine contributes to tumor angiogenesis by activating the A2B adenosine receptor on MDSCs. This effect and accumulation of MDSCs are blocked by the addition of A2B receptor antagonist PSB1115 [70].

Overall, the mechanisms by which the upregulation of the above-mentioned metabolic pathways regulate MDSC maturation, infiltration, accumulation, and function is currently unknown in HGG and warrants further investigation. However, the promising results obtained by inhibiting these pathways in other cancer types provide the necessary impetus to try those in the context of high-grade gliomas.

## 5. Immunosuppression Mediated by MDSCs

**Figure 4:**
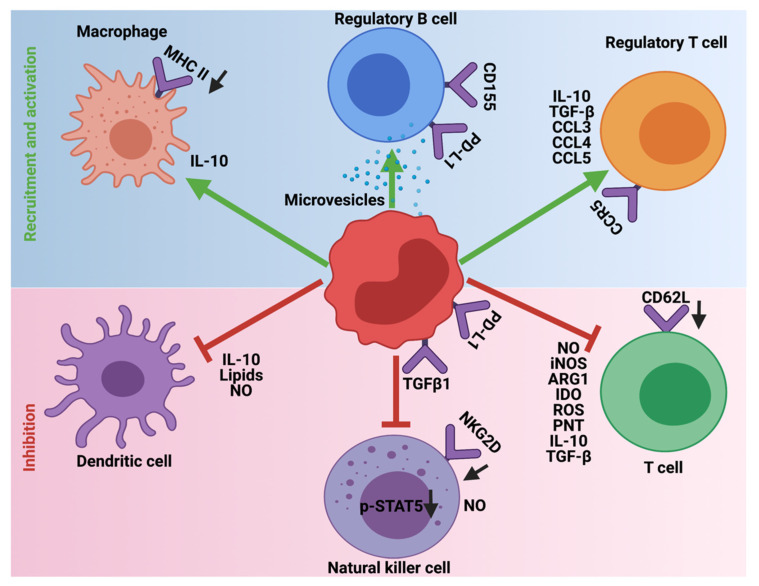
Immunosuppressive role of MDSCs in HGG. MDSCs employ different mechanisms to inhibit the cytotoxic and antigen presentation functions of T-cells, Natural killer cells, and Dendritic cells. Additionally, MDSCs promote the recruitment and activation of immune-suppressive Tregs, Bregs, and M2 macrophages. The downward arrows in black indicate downregulation.

In steady-state or homeostatic conditions MDSCs lack any immunosuppressive activity. This is due to the shared characteristics of MDSCs with neutrophils and monocytes. However, in pathological diseases, inflammation, obesity, sepsis, and cancer, immunosuppression is one of the major functions orchestrated by MDSCs. To this end, MDSCs employ a variety of mechanisms to inhibit the function of T-cells, NK, dendritic cells (DC), and macrophages and promote the recruitment of Tregs and immune-suppressive B-cells to support the development and progression of HGG (Figure 4). Furthermore, MDSCs generate and maintain an immunosuppressive TME and limit the efficacy of cancer immunotherapies.

MDSCs suppress T-cell functions using different mechanisms. Specifically, in rat glioma models, MDSCs produce NO, iNOS, ARG1, and IDO to suppress T-cell proliferation and induce T-cell apoptosis [71]. Furthermore, ARG1 and IDO catabolize extracellular arginine and tryptophan, the essential amino acids required for T-cell activation and function [72]. Consistent with this, MDSCs express xc-transporter and sequester cysteine, another essential amino acid that is vital for T-cell proliferation and activation [73]. MDSCs also produce ROS, peroxynitrite (PNT), and secrete anti-inflammatory cytokines such as IL-10 and TGF-β to interfere with T-cell cytotoxic function. Briefly, ROS and PNT cause the nitration of the T-cell receptor, which then prevents its binding to peptide-major histocompatibility complex [74,75]. IL-10 downregulates the expression levels of L-selectin (CD62L) on naïve T-cells to prevent their migration to the tumor site [76], whereas TGF-β promotes the differentiation of TH17 cells (a subset of CD4 T-cells) into Tregs [77]. Finally, MDSCs upregulate the expression of immune checkpoint molecules such as PD-L1 that interacts with PD-1 on T-cells and causes their exhaustion [78]. 

Similar to T-cells, NK cells have the tremendous potential to eliminate glioma cells [79]. However, the presence of MDSCs in HGG decreases the cytotoxicity of NK cells. For instance, NO produced by MDSCs inhibits the Fc receptor-mediated NK function in a contact-independent fashion [80]. Further, membrane-bound TGFβ1 on MDSCs reduces the cytotoxic function of NK cells by downregulating the expression of INFγ and activating receptor NKG2D [81]. Importantly, NK cells that are deficient for TGF-β were immune to the MDSC-mediated suppressive effects [82]. Lastly, CD11b+ Gr1+ MDSCs were found to accumulate in the spleens of tumor-bearing mice and there it suppresses the perforin production by NK cells through the modulation of JAK-STAT (decreasing p-STAT5) signaling pathway [83]. 

The frequencies of matured dendritic cells (DCs) decrease as the MDSC numbers increase in tumors via MyD88-NF-κB signaling [84]. Furthermore, MDSCs secreted IL-10 in the TME inhibit the IL-12 induced T-cell stimulatory function of DCs. In line with these findings, the Il-12 treatment induces the expansion of CD11c (a marker for DCs) in splenocytes of tumor-bearing mice and differentiation of M-MDSC to DCs [85]. Moreover, NO produced by MDSCs in the TME hampers the antigen presentation from DCs to CD4+ T-cells and this effect was reversed by the addition of iNOS inhibitors [86]. Moreover, MDSCs deliver lipid bodies (LBs) enclosed with oxidatively truncated lipids to DCs. These LBs then interact with heat shock protein 70 (HSP70) and prevent the expression of peptide-MHC class 1 expression on the surface of DCs, thereby impairing the antigen cross-presentation function of DCs to T-cells [87].

Along with inhibiting the function of NK, DC, and T-cells as described above, MDSCs contribute to the immunosuppressive TME in glioma by recruiting alternatively activated or M2 macrophages, Bregs, and Tregs. The cross-talk between MDSCs and tumor-associated macrophages is well-documented in glioma [88]. MDSCs reduce the antigen presentation function of macrophages by reducing the MHC-class II expression on them. Furthermore, the presence of MDSCs in tumors reduces the anti-inflammatory M1 macrophages and expands pro-tumor M2 macrophages [89]. Moreover, MDSCs secrete IL-10 to prevent macrophages from generating an inflammatory cytokine, IL-12 that activates CD4+ T-cells, DCs, and NK cells [90]. In addition, hypoxic conditions in the TME environment recruit MDSCs into the tumor site, which then differentiates into tumor-associated macrophages (TAMs) [91].

MDSCs secrete IL-10, INFβ, and TGF-β to promote the differentiation of naïve CD4+ T-cells into Tregs and provide a suppressive TME that is conducive for the expansion of Tregs [92]. The cytokines, chemokines (CCL3, CCl4, CCL5) secreted by MDSCs directly recruit Tregs via CCR5 into the TME [93]. Recent studies suggest that direct interaction between MDSCs and Tregs in mice and human tumors suppressed CD8+ T-cell effector responses and promoted their apoptosis [94]. Depletion of Tregs using anti-CD25 mAb results in the accumulation of non-immune suppressive macrophages and granulocytes in mouse HGG glioma models [95]. 

MDSCs use the recruited regulatory B-cells (Bregs) in the HGG microenvironment to perform their immune-suppressive functions. Bregs account for about 40% of the immune-infiltrating cells in GBMs. In the TME, Bregs express elevated levels of PD-L1 and CD155 as well as secrete immunosuppressive cytokines TGF-β and IL-10 to prevent the cytotoxic activity of CD8+ T-cells. One of the mechanisms by which Bregs upregulate immune checkpoint molecules and cytokines is through the uptake of MDSC-derived microvesicles containing PD-L1. Consistent with these findings, depletion of B-cells using the anti-CD20 monoclonal antibody increased the survival. Therefore, a dialog between MDSCs and B-cells plays an important role in GBM progression [96]. On the other hand, B-cells are shown to induce T-cell mediated anti-tumor immunity by acting as antigen-presenting cells (APCs) [97]. Moreover, the presence of B-cells correlates with reduced tumor growth, invasion, and increased survival in GBM patients [98]. Thus, the exact role of B-cells in HGG is still not clear and remains to be determined. 

Taken together, MDSCs are one of the key players in avoiding immune surveillance in HGG. To achieve this, MDSCs deploy different strategies to prevent the cytotoxic functions and maturation of T-cells, NK cells, and DCs. Additionally, they provide a favorable TME to support the recruitment and inhibitory functions of M2 macrophages, Tregs, and Bregs. Whether some of these outlined mechanisms contribute to the immunosuppression by MDSCs in HGGs remain to be investigated. 

## 6. MDSCs Induced Therapeutic Resistance

**Figure 5:**
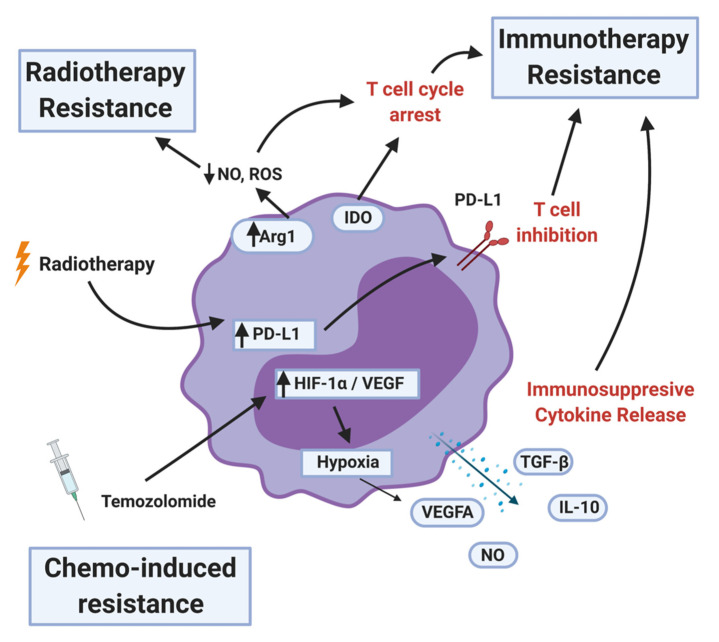
MDSC mechanisms of therapy resistance. MDSCs contribute to radiotherapy resistance by depleting nitric oxide (NO) and reactive oxygen species (ROS) and upregulating programmed death ligand-1 (PD-L1) after the treatment. MDSCs inhibit immunotherapy by arresting or inhibiting T-cell function, recruiting T-regulatory cells, and secreting immunosuppressive cytokines and other factors. Less is known about chemotherapy resistance, but certain MDSC populations can be a proxy for therapy response. MDSCs that survive the initial temozolomide treatment upregulates hypoxia-inducible factor-1 alpha (HIF-1α) and vascular endothelial growth factor (VEGF), resulting in pro-tumor angiogenesis.

MDSCs levels in the peripheral blood as well as at the tumor site are used as a biomarker to predict if the existing standard-of-care therapies would work in cancer patients. High levels of MDSCs are often correlated with chemo, radiation, and immunotherapy resistance (Figure 5). 

MDSCs contribute to chemotherapy in two ways: Initial chemotherapy resistance and induced immunosuppression following chemotherapy. Limited information on which subsets of MDSCs contribute to chemoresistance is available until now. Furthermore, the underlying mechanisms of how MDSCs contribute both directly and indirectly to chemotherapy resistance are not well understood.

MDSCs increase the recruitment of suppressive T-regulatory cells to the tumor site, which results in both radiotherapy and chemotherapy resistance [99]. Several subpopulations of MDSCs have been shown to play important roles in their respective cancer models while also serving as a proxy for response to chemotherapy. Glioma patients have shown elevated levels of MDSCs expressing S1008/9 along with increased arginase production [100]. Similarly, S100A9(+) inflammatory monocytes in patients with non-small cell lung cancer (NSCLC) are shown to suppress T-cells by production of arginase, iNOS, and the IL-13/IL-4Rα axis. The amount of these inflammatory monocytes is associated with poor response to chemotherapy [101].

In many clinical trials, increased immunosuppression is, unfortunately, a side effect of chemotherapy treatments. Temozolomide is one of the common chemotherapeutic agents used for treating glioblastoma. While it does have anti-tumor effects and increased patient survival, it can also induce increased expression of hypoxia-related genes HIF-1α and VEGF [102]. These factors promote a cascade of pro-tumor pathways including angiogenesis and cell repair (Figure 5). Systemic temozolomide administration also results in immunodepletion, which can be an advantage when used in combination with immunotherapies but presents another hurdle for establishing anti-tumor activity in lymphocytes. In lung cancer mouse models, carboplatin potentiates an MDSC dependent pathway that triggers resistance through upregulated VCAM/RANTES and activating of IL13/33 pathways [103]. These chemo-induced MDSCs perform their immune suppressive role by promoting the accumulation of IL-10 producing CD4+/Foxp3+ Tregs (regulatory T-cells) [103]. 

The application of ionizing radiation, known as radiotherapy (RT), is largely based on its cytocidal activity combined with the ability to selectively target tumors. Radiotherapy facilitates anti-tumor immunity both locally and at distant, non-radiated sites (abscopal effect) [104]. RT can reduce MDSC levels in the TME, but MDSCs are relatively short-lived and frequently replenished by circulating MDSCs, which are often unaffected by RT and elevated in glioma patients [48]. High Arginase-1 (Arg1) expression by subsets of MDSCs deplete local levels of nitric oxide and other reactive oxidative species which confer RT sensitivity under hypoxic conditions (Figure 5) [105,106].

RT can also promote the tumor expression of known checkpoint inhibitors such as PD-L1 [106]. As a result of this increased immunosuppression, many ongoing studies seek to combine the anti-tumor effects of RT while using checkpoint inhibitors to mitigate any potential immunosuppressive side effects. An alternative approach is to modify the intensity and schedule of RT. Recent studies have utilized ablative hypofractionated RT (AHFRT) rather than the conventional fractionated RT (CFRT). Compared with CFRT, AHFRT significantly inhibits tumor growth and reduces recruitment of MDSCs, most likely through downregulation of VEGF and decreasing tumor hypoxia [107]. However, in the context of brain cancers, high doses of radiation can have detrimental side effects. Utilizing AHFRT plus temozolomide in glioblastoma found no increase in the overall survival [108]. Currently, AHFRT is an accepted standard of care only in patients with poor performance status or advanced age [109].

MDSCs represent a large obstacle to the immune checkpoint blockade due to their direct suppression and indirect induction of immunosuppressive phenotypes in other immune cells in the tumor microenvironment. In glioblastoma, MDSCs are influenced by tumor cells, including cancer stem cells, towards their immunosuppressive phenotype both locally and systemically [48,100].

As discussed earlier (see immunosuppression mediated by MDSCs), MDSCs secrete immunosuppressive cytokines and other factors such as TGF-β, IL-10, NO, and ROS. They also upregulate proteins that induce T-cell dysfunction: Arg1, IDO1, and PD-L1 (Figure 5). A further consequence of MDSCs is the induction of immunosuppressive phenotypes in other immune cell types in the TME. MDSCs promote the differentiation of Treg cells [110]. Tregs are potent suppressors of both the adaptive and innate immune system [111]. MDSCs contribute to increased NK cell dysfunction [81] and T-cell exhaustion by inducing T-cell expression of TIGIT, CTLA-4, TIM3, LAG3, and CD160 [112,113]. Whether these effects seen in other tumor types translate to HGG remains to be investigated.

The result of MDSCs in the TME is a self-reinforcing network of immune suppression that supports tumor proliferation. As a result of these known suppressive mechanisms, it is no surprise that the presence of MDSCs can affect the efficacy of IC therapy. In a model of metastatic melanoma, MDSC levels could be used as a predictor of ipilimumab (anti-CTLA4 monoclonal antibody) treatment efficacy [114]. Future treatment models may need to include an anti-MDSC component. Promising research in mouse models has shown rejection of tumor and immunologic memory when combining immune checkpoint inhibitors (nivolumab, avelumab, and ipilimumab) with MDSC targeted therapies as discussed below [115].

## 7. Strategies for Therapeutic Targeting of MDSCs 

**Figure 6:**
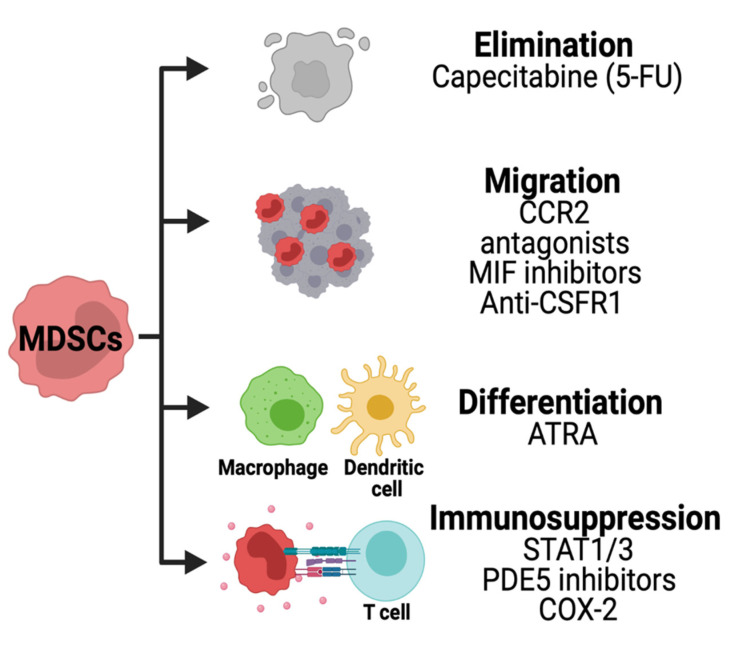
Strategies for targeting MDSCs in HGG. Chemotherapies are being used to deplete MDSCs in solid tumors. The migration of these cells is inhibited using CCR2, macrophage inhibitory factor (MIF) or CSFR1 inhibitors. Other therapies are used to decrease the immunosuppression pathways and promote MDSCs differentiation.

Current therapies for HGG have failed until now, in part due to the immune suppressive functions of MDSCs as described above. Henceforth, several approaches are being pursued to therapeutically target MDSCs. Standard of care and current immunotherapies for brain tumors have failed, in part due to the immunosuppressive function of MDSCs. Several strategies are being developed to therapeutically target MDSCs in solid cancers (Figure 6).

The immunosuppressive activity of MDSCs can be disrupted by inhibiting stem cell factors primarily produced by tumor cells. The JAK2-STAT3 pathway is known to regulate immune responses via cytokines. JAK2 phosphorylation and activation cause translocation of STAT proteins into the nucleus regulating anti-apoptotic and pro-proliferative genes in MDSCs. For instance, STAT1 controls arginase-1 (ARG1) and iNOS in M-MDSCs, while STAT3 is involved in proliferation and reactive oxygen species (ROS) formation in G-MDSCs [116,117]. A STAT3 inhibitor has been identified from the herb Curcuma longa Linn commonly known as curcumin. Studies have shown the benefits of curcumin in brain tumor cells, decreasing their proliferative and antiapoptotic capacity [118,119]. However, the effects of STAT3 inhibition in MDSCs in the setting of brain tumors remain unexplored to this date. 

Phosphodiesterase 5 (PDE5) is another promising therapeutic targeting immunosuppressive pathway in MDSCs. PDE5 is a hydrolase that regulates the NO/cyclic guanosine monophosphate (cGMP) pathway [120]. Mice with carcinomas treated with sildenafil, a PDE5 inhibitor, had downregulation of ARG1, NOS2, and IL4Rα in MDSCs [121]. Preoperative administration of sildenafil has been shown to reduce levels of ARG1 and ROS without disturbing the accumulation of gMDSCs increasing antitumoral NK cell cytotoxicity towards murine melanoma tumors [122]. In the phase 2 clinical trial, patients treated with tadalafil, another PDE5 inhibitor, resulted in a reduction of MDSCs in the blood and tumor possibly by decreasing pro-survival IL4Rα on MDSCs [123]. Inhibition of PDE5 by sildenafil has been shown to increase chemotherapeutic response in medulloblastoma cells [124]. Preclinical studies using syngeneic mouse models and clinical trials should be performed to assess the effects of PDE5 inhibitors in brain cancer patients.

Tumor cells producing PGE2 can promote MDSCs immunosuppression by activating TGFβ through PGE2 receptors Ep2 and Ep4 found in monocytes [125]. The generation of PGE2 by tumor cells is partially regulated by cyclooxygenase-2 (COX-2). The COX-2 inhibitors acetylsalicylic acid (ASA) and celecoxib decreased systemic levels of PGE2 and MDSCs immunosuppression in the murine glioma model [126].

The release of colony-stimulating factor 1 (CSF-1) from tumors allows CSF-1R+ myeloid cells to proliferate and differentiate into MDSCs. In proneural gliomas, CSF-1R inhibition initially increased the survival of mice and decreased M2-like TAMs [127]. However, gliomas developed resistance to CSF-1R through the PI3K mediated release of insulin-like growth factor 1 (IGF-1) [128]. In a concluded clinical trial, the oral administration of CSF-1R inhibitor PLX3397 was well tolerated but not efficient to treat GBM patients [129]. These studies have shown partial efficacy of CSF-1R inhibition and the potential benefit of combining it with other therapies to overcome resistance.

The migration and accumulation of MDSCs into glioma TME has been reported to be mediated by CCL2. Glioma tumor cells release CCL20 and induce CCL2 production in TAMs and GAMs attracting MDSCs into TME [41]. Accumulation of MDSCs in glioma was abrogated in CCR2 deficient mice [41]. The use of CCR2 antagonist CCX872 reduced intratumoral MDSCs and enhanced the anti-PD-1 treatment in GBM-bearing mice [130]. In addition to CCL2, the macrophage inhibitory factor (MIF) is secreted by glioma cells and regulates MDSCs recruitment into TME [131]. Sulforaphane, a MIF inhibitor derived from broccoli sprouts, decreased levels of MDSCs in monocytes cultured with hypoxic glioma conditioned media [132]. In vivo, sulforaphane induces apoptosis of glioma flank tumors [133]. Ibudilast is another MIF inhibitor shown to target M-MDSCs through the CD74 receptor decreasing the release of MCP-1 [37].

Low levels of chemotherapies can be used to deplete MDSCs. The use of 5-FU on mice bearing GBM was able to decrease tumor-derived MIF, resulting in decreased arginase-1 expression in MDSCs [131]. Similarly, a phase 0/1 clinical trial was conducted showing that a combination of capecitabine (an orally 5-FU drug) and bevacizumab (anti-angiogenic antibody) were able to decrease circulating MDSCs in patients with recurrent GBM [134]. These studies suggest the benefit of combining chemotherapies with other targeted therapies.

Other therapeutic approaches are being developed to promote MDSCs differentiation dampening their pro-tumor activity. MDSCs isolated from cancer patients were differentiated into dendritic cells (DC) using ATRA and GM-CSF (Al). In MDSCs, ATRA causes upregulation of glutathione synthase resulting in the accumulation of glutathione and reduction of ROS [135]. In brain cancer cells (glioma and medulloblastoma), ATRA treatments cause cell growth arrest [136,137,138]. However, the combination of ATRA with epigenetic drugs SAHA and 5-AZA exacerbated tumor growth in a glioma xenograft mouse model [139]. The effects of ATRA in MDSCs of brain tumors remain to be determined.

Most treatments targeting MDSCs directly or indirectly have been evaluated in other solid tumors than in brain cancers (Table 1). Studies have shown the effects of some of these MDSCs targeted therapies in brain tumor cells but not in MDSCs. Future work should be done to fully understand the effects of MDSC targeted therapies in the immune microenvironment of brain tumors.

## 8. Conclusions, Open Questions, and Future Perspectives

MDSCs are one of the abundant immune suppressive cells that are present in peripheral blood as well as at the tumor site in HGG patients. Recent observations have started to inform that similar to cancer cells, MDSCs alter their metabolic pathways to adapt and thrive in extreme conditions offered by the glioma TME. Furthermore, a growing body of evidence as outlined in this review highlight the profound effects of MDSCs in inducing immunosuppression, therapeutic resistance, and how targeting them in mouse and rat GBM models is beneficial for increasing the survival of these animals. However, so far clinical approaches aimed at targeting MDSCs in human cancer patients have not lived up to the expectations. The reasons for this are due to the incomplete understanding of MDSC phenotypic and functional heterogeneity and the strategies utilized by MDSCs to resist chemo, radiation, and immunotherapies. Some of this can be addressed in preclinical tumor models using state-of-the-art technologies such as single-cell RNA-sequencing (scRNA-seq), nanostring, mass cytometry, multiplexed immunohistochemistry, single-cell assay for transposase accessible chromatin using sequencing (scATAC-seq), and whole-genome sequencing.

Several outstanding questions remain: First, what are the true phenotypic markers that separate MDSC subsets from monocytes and neutrophils. Second, what are the molecular mechanisms that govern the generation, recruitment, and accumulation of MDSCs in HGG? Third, what metabolic programs are altered in MDSCs? Are these alterations common or different in M-MDSCs compared to PMN-MDSCs? Finally, whether targeting MDSCs using different approaches (as mentioned in Strategies for therapeutic targeting of MDSCs) alone as monotherapy is sufficient for inducing significant and long-term durable responses in the clinic or a combination with other treatments is needed? If combination therapy is required then the details regarding the type, sequence, and timing of these treatments have to be worked out. Finding the answer to these questions in the upcoming years will result in novel and efficient treatment options that would improve the survival of high-grade glioma patients by precisely targeting MDSCs. 

## Figures and Tables

**Table 1:** Clinical trials targeting MDSCs directly or indirectly in HGGs.

Tumor Type	Drug	Target	Combination Strategy	Status	Clinical Trial Identifier	Effects on Clinical Outcome	References
Glioblastoma	Capecitabine	Tumor cells	Bevacizumab	Active, recruiting	NCT02669173	Reduction in MDSC frequencies and increase in cytotoxic immune cells is observed in GBM patients	[134]
Glioblastoma	Atezolizumab	PDL1	Ipatasertib	Active, recruiting	NCT03673787	Early results show reduction of regulatory T-cells and increase in effector T-cells in solid tumor patients	[140]
Glioblastoma	IMA 950 (multi tumor-associated peptides vaccine)	Immune cells	Vaccine adjuvant Poly ICLC	Completed	NCT01920191	Sustained CD8 and CD4 T-cell responses leads to an increase in medial survival of GBM patients by 19 months	[141,142]
Advanced solid tumors (including malignant glioma)	Cabiralizumab	CSFR1	Nivolumab	Completed	NCT02526017	Decreases circulating monocytes in solid tumor patients. Some adverse effects such as increase in creatine kinase and serum liver enzymes are reported	[143,144]

## Data Availability

Not applicable.
